# Human leukocyte antigen class I and class II alleles are associated with susceptibility and resistance in borderline leprosy patients from Southeast Brazil

**DOI:** 10.1186/s12879-015-0751-0

**Published:** 2015-01-21

**Authors:** Fabiana Covolo de Souza-Santana, Elaine Valim Camarinha Marcos, Maria Esther Salles Nogueira, Somei Ura, Jane Tomimori

**Affiliations:** Immunogenetics Laboratory, Instituto Lauro de Souza Lima, Rod. Cte João Ribeiro de Barros, km 225/26, Bauru, SP CEP: 17039-800 Brazil; Immunology Laboratory, Instituto Lauro de Souza Lima, Rod. Cte João Ribeiro de Barros, km 225/26, Bauru, SP CEP: 17039-800 Brazil; Department of Education and Research, Instituto Lauro de Souza Lima, Rod. Cte João Ribeiro de Barros, km 225/26, Bauru, SP CEP: 17039-800 Brazil; Department of Dermatology, Federal University of São Paulo, UNIFESP, Av. Borges Lagoa, 598, São Paulo, SP CEP: 04038-000 Brazil

**Keywords:** Leprosy, Borderline, HLA alleles, Genetics, *Mycobacterium leprae*

## Abstract

**Background:**

Evidence suggests that human leukocyte antigen (HLA) alleles influence the host immune response against *Mycobacterium leprae*. However, the association between HLA alleles and borderline (B) leprosy has not been studied. The aim of this study was to determine whether HLA class I and II molecules are associated with susceptibility or resistance to B leprosy including borderline-tuberculoid (BT), borderline-borderline (BB), and borderline-lepromatous (BL).

**Methods:**

DNA was obtained by the *salting-out* technique from the blood samples of 202 patients with B leprosy and 478 control subjects. HLA class I (A*, B*, and C* loci) and class II (DRB1* and DQB1* loci) genotypes were determined by polymerase chain reaction amplification and reverse hybridization with sequence-specific oligonucleotide probes and sequence-specific primers.

**Results:**

The case-controlled analysis results showed a significant association between B leprosy and HLA-C*05 (5.94% *vs*. 14.02%; *p =* 0.002, OR = 0.38, 95% CI = 0.20–0.73, *pc =* 0.032) and HLA-DRB1*07 (16.34% *vs*. 26.77%; *p =* 0.003, OR = 0.53, 95% CI = 0.3–0.8, *pc =* 0.039). A protective association was observed between BL leprosy and HLA-DQB1*02 (18.18% *vs*. 39.53%; *p* = 0.005, OR = 0.34, 95% CI = 0.15–0.75, *pc* = 0.025). In reactional patients, a significant association was observed between HLA-B*15 (28.72% *vs.* 12.76%; *p* = 0.011, OR = 2.75, 95% CI = 1.30–5.85, *pc* = 0.352) and predisposition to reversal reaction. Haplotype analysis showed that A*02-B*07-C*07-DRB1*15-DQB1*06 (2.97% *vs.* 1.04%; *p* = 0.015) and A*02-B*40-C*03-DRB1*13-DQB1*06 (1.73% *vs.* 0.10%; *p* = 0.0011) were associated with susceptibility to the B form. The presence of the HLA-DRB1*02 or HLA-DRB1*03/HLA-DQB1*01 haplotypes in B patients (22.05% *vs.* 33.0%; *p =* 0.005) suggested the involvement of these haplotypes in this clinical form of the disease.

**Conclusions:**

The results indicate the involvement of HLA class I and class II molecules in B leprosy and reversal reactions; it also suggest a role for HLA in polarization of the disease in this group of patients.

**Electronic supplementary material:**

The online version of this article (doi:10.1186/s12879-015-0751-0) contains supplementary material, which is available to authorized users.

## Background

Leprosy is a chronic infectious disease caused by *Mycobacterium leprae*, an obligate intracellular pathogen that has a preference for Schwann cells in the peripheral nervous system and macrophages in the skin. The host cellular immune response is the main mechanism of resistance to the parasite [1]. The balance in the immune response of the host is strongly correlated with the clinical spectrum of the disease, which includes 5 distinct clinical forms, with 2 extremes, tuberculoid and lepromatous leprosy. Tuberculoid leprosy (TT) is characterized by resistance to bacilli proliferation due to a predominant Th1 response, which results in a strong cellular immune response. At the other extreme, lepromatous leprosy (LL) is characterized by the spread of bacilli due to a weak cellular immune response and a predominant Th2 response, which induces the production of antibodies [2].

Between these 2 extremes are intermediate or borderline (B) forms, which are considered unstable forms because they exhibit manifestations between TT and LL. The intermediate forms are borderline-tuberculoid (BT), borderline-borderline (BB), and borderline-lepromatous (BL), and these 3 forms show a gradual diminution in the cellular immune response and resistance to the bacilli [2]. Some authors [3,4] have suggested that these clinical forms can either oscillate or remain stable, and in the latter case, they are mutually incompatible. In B leprosy, so-called leprosy reactions occur. The type 1 reaction or reversal reaction (RR) affects mainly BL, BB, and BT patients and is associated with spontaneous acquisition of cellular immunity against *M. leprae* antigens [5,6]. The type 2 reaction or erythema nodosum leprosum (ENL) occurs in multibacillary patients (BL and LL) with low cellular immune responses and is related to the deposition of immune complexes in response to *M. leprae* antigens [5,6]. These type 1 and type 2 reactions affect 30% to 50% of patients, respectively, and cause great suffering and sequelae [5-7].

The influence of genetic factors on the ability of individuals to resist diseases, including leprosy, has long been studied [8,9]. The susceptibility phenotype of *M. leprae* infection is complex and is influenced by numerous factors in both the host and parasite as well as by environmental conditions [10].

The histocompatibility leukocyte antigen (HLA) complex, located on chromosome 6p21, is the most polymorphic genetic system in mammals [11]. The encoded proteins are involved in antigen presentation to T-cell receptors (TCRs), which triggers a specific immune response [12]. In leprosy, studies of the HLA complex have largely been focused on elucidating the mechanisms related to individual susceptibility/resistance and disease prognosis. Studies of HLA class I (HLA-A, HLA-B, and HLA-C) have been conducted in various populations [13-18]. For HLA class II (HLA-DR and DQ), the most consistent findings are that HLA-DR2 (subtypes HLA-DRB1*15 and HLA-DRB1*16) and HLA-DQ1 (subtypes HLA-DQB1*05 and HLA-DQB1*06) are associated with the TT and LL clinical forms, respectively [19-27], and are also associated with leprosy *per se* [28-34]. To confirm these previous findings, 2 genome-wide linkage analyses was done and identified chromosome 6p21 as a major leprosy susceptibility locus in the HLA gene cluster [35,36].

Based on these previous results, and because the involvement of HLA in borderline (B) leprosy and leprosy reactions have not been described, the aim of this study was to investigate the possible association of HLA class I and II alleles and haplotypes with B leprosy forms, including BT, BB, and BL and leprosy reactions in a sample population from São Paulo State, Brazil.

## Methods

### Patients and controls

This study included 202 patients diagnosed with leprosy classified as B type by a medical team in the Dr. Diltor V. A. Opromolla Department of Dermatology at Lauro de Souza Lima Institute (ILSL) in Bauru, São Paulo State, Southern Brazil. For the purpose of classification and inclusion in the study, we searched medical records to determine the details regarding clinical classification in addition to the complementary exams given at diagnosis, such as histopathology, Mitsuda reaction, and slit skin smear. The study patients were followed for 1 year to assess the presence and classification of leprosy reactions. Of the 202 patients, 143 (70.8%) were men, and the ages of the patients ranged from 7 to 86 years, with a median age of 51 years. According to the clinical, immunological, baciloscopic, and histopathological indexes proposed by Ridley and Jopling [2], 88 patients were classified as having BB, 70 were classified as having BT, and 44 were classified as having BL. Ninety four patients (46.5%) had reversal reactions, 10 patients (4.95%) had an erythema nodosum leprosum (ENL) reactions at diagnosis and/or during the course of the disease, and 94 (46.5%) had no leprosy reaction. For 4 of the 202 patients (2.0%), no leprosy reactions were reported during the 1-year follow-up.

The control group consisted of 478 unrelated, healthy individuals of the same ethnicity from the same geographic region with no history of leprosy or other disease associated with HLA, who underwent HLA typing in the Immunogenetics Laboratory of ILSL. Of the 478 control individuals, 200 (41.8%) were men, and the ages of the controls ranged from 20 to 68 years, with a median age of 37 years.

This was a retrospective case-controlled study in which we used DNA samples obtained from patients and controls that were deposited in the DNA bank of the Immunogenetics Laboratory of ILSL. All subjects gave informed consent to participate in the study. The Ethics Committees of UNIFESP (1048/10) and ILSL (039/2010) approved this study.

### DNA extraction

Peripheral venous blood samples were collected from patients into tubes containing anticoagulant EDTA, and DNA was extracted from buffy coats by using the salting-out technique [37]. DNA samples were stored in −80°C until use.

### HLA class I and class II typing

HLA class I (A*, B*, and C* loci) and class II (DRB1* and DQB1* loci) genotypes were determined at low resolution by using the Luminex 100 xMAP flow cytometry dual-laser system to quantify fluorescently labeled oligonucleotides attached to color-coded microbeads (One Lambda®, Canoga Park, CA, USA) according to the manufacturer’s instructions. Briefly, the HLA region was amplified by polymerase chain reaction-sequence specific oligonucleotide (PCR-SSO) using a specific primer. The PCR product was denatured and hybridized to complementary DNA probes attached to fluorescently tagged microbeads. The biotinylated reaction product was detected through binding to streptavidin conjugated to R-phycoerythrin, which resulted in fluorescence emission. A flow analyzer, the LabScan™ 100, was used to measure the fluorescence intensity of each microsphere. The data were analyzed with HLA Fusion software (One Lambda®, Canoga Park, CA, USA).

### Statistical analysis

Allele frequencies were determined by direct counting. The distribution of haplotype frequencies were estimated using the Expectation Maximization (EM) algorithm. Hardy-Weinberg equilibrium was tested to confirm the distribution of allele frequency. Both tests were performed by using Arlequin software, version 3.0 [38].

Case-controlled analyses of HLA alleles and B clinical forms (BB, BT, and BL) were performed using the chi-square test or Fisher’s exact test with Graph Pad (http://http://www.graphpad.com/quickcalcs/contingency1.cfm). P values less than or equal to 0.05 were considered statistically significant. Bonferroni correction was applied when the p values were significant (by multiplying the p values by the number of HLA alleles being tested), and pc values less than or equal to 0.05 were considered significant. Odds ratios were calculated with a 95% confidence interval (95%CI) using SISA when the p values were significant.

## Results

The possible associations between HLA class I and class II alleles and B leprosy were assessed by comparing the frequencies of these alleles in patients with B leprosy to those in healthy controls and by comparing the frequencies in patients with different B clinical forms (BB, BT, and BL). Details of the analysis are shown in Additional file 1 and Additional file 2.

### B leprosy and healthy controls

An increased frequency of HLA-B*07 was observed in B leprosy patients (18.81% *vs.* 12.78%; p = 0.043, OR = 1.58, 95% CI = 1.02–2.5, pc = 1.37). HLA-B*53 (6.43% *vs.* 2.93%; p = 0.050, OR = 2.28, 95% CI = 1.05–4.94, pc =1.60) and HLA-C*16 (10.4% *vs.* 5.44%; p = 0.030, OR = 2.02, 95% CI = 1.10–3.67, pc = 0.48) were also more frequent in B leprosy patients than in healthy controls. A decreased frequencies of HLA-B*49 (0.99% *vs.* 6.07%; p = 0.002, OR = 0.15, 95% CI = 0.04–0.65, pc = 0.064), HLA-B*50 (1.98% *vs.* 6.28%; p = 0.019, OR = 0.30, 95% CI = 0.10–0.87, pc = 0.608), HLA-C*05 (5.94% *vs.* 14.02%; p = 0.002, OR = 0.38, 95% CI = 0.20–0.73, pc = 0.032), and HLA-DRB1*07 (16.34% *vs.* 26.77%; p = 0.003, OR = 0.53, 95% CI = 0.3–0.8, pc = 0.039) were observed in the group of patients. After p-value correction, the associations that remained significant were HLA-C*05 and HLA-DRB1*07, suggesting that these alleles provide protection against B leprosy, whereas HLA-B*49 only showed a trend toward a protective effect. Table 1 shows the results of the HLA allele association analysis in B leprosy patients and the healthy controls.

**Table 1:** **HLA alleles showing statistically significant differences in frequency between B leprosy patients and healthy controls**

**B leprosy**	**HLA allele**	**Patients (N = 202)**		**Controls (N = 478)**		**p**	**pc**	**OR**	**IC**
		**N**	**%**	**n**	**%**				
	B*07	38	18.81	61	12.78	0.043	1.37	1.58	1.02–2.50
	B*49	2	0.99	29	6.07	0.002	0.064	0.15	0.04–0.65
	B*50	4	1.98	30	6.28	0.019	0.608	0.30	0.10–0.87
	B*53	13	6.43	14	2.93	0.050	1.60	2.28	1.05–4.94
	C*05	**12**	**5.94**	**67**	**14.02**	**0.002**	**0.032**	**0.38**	**0.20–0.73**
	C*16	21	10.4	26	5.44	0.030	0.48	2.02	1.10–3.67
	DRB1*07	**33**	**16.34**	**128**	**26.77**	**0.003**	**0.039**	**0.53**	**0.30–0.80**

N: number of individuals, n: number of alleles (2n), %: allele frequency, p: Fisher’s exact test (p ≤ 0.05), pc: corrected p value, OR: odds ratio, CI: confidence interval, boldface: significant association after Bonferroni´s correction.

### BB, BT, and BL leprosy and healthy controls

Analysis of the HLA allele frequency showed an increased frequency of HLA-B*58 (11.36% *vs.* 5.03%; p = 0.028, OR = 2.42, 95% CI = 1.11–5.27, pc = 0.896) and HLA-C*12 (21.59% *vs.* 13.39%; p = 0.050, OR = 1.78, 95% CI = 1.00–3.15, pc = 0.70) and a decreased frequency of HLA-B*49 (0.0% *vs.* 6.07%; p = 0.014, OR = 0.09, 95% CI = 0.005–1.42, pc = 0.448) and HLA-C*05 (5.68% *vs.* 14.02%, p = 0.035, OR = 0.37, 95% CI = 0.14–0.94, pc = 0.490) in patients with BB leprosy. After correction, these results did not remain significant. The results of the association analysis between BB leprosy and the healthy control group are shown in Table 2.

**Table 2:** **HLA alleles with statistically significant differences in BB, BT, and BL patients and healthy controls**

**BB**	**HLA allele**	**Patients (N = 88)**		**Controls (N = 478)**		**p**	**pc**	**OR**	**IC**
		**N**	**%**	**n**	**%**				
	B*49	0	0.00	29	6.07	0.014	0.448	0.09	0.005–1.42
	B*58	10	11.36	24	5.03	0.028	0.896	2.42	1.11–5.27
	C*05	5	5.68	67	14.02	0.035	0.49	0.37	0.14–0.94
	C*12	19	21.59	64	13.39	0.050	0.70	1.78	1.00–3.15
**BT**		**Patients (N = 70)**		**Controls (N = 478)**					
	A*33	8	11.43	25	5.23	0.056	1.12	2.34	1.01–5.41
	B*40	2	2.86	50	10.46	0.047	1.50	0.25	0.06–1.06
	B*49	0	0.00	29	6.07	0.039	1.24	0.11	0.006–1.79
	B*53	6	8.57	14	2.93	0.031	0.992	3.11	1.15–8.37
	C*05	3	4.28	67	14.02	0.020	0.28	0.27	0.08–0.89
	C*16	9	12.86	26	5.44	0.031	0.434	2.56	1.15–5.73
	DRB1*03	20	28.57	85	17.78	0.049	0.637	1.85	1.05–3.27
**BL**		**Patients (N = 44)**		**Controls (N = 478)**					
	B*53	4	9.09	14	2.93	0.056	1.792	3.31	1.04–10.54
	DRB1*07	5	11.36	128	26.77	0.028	0.364	0.35	0.13–0.91
	DQB1*02	**8**	**18.18**	**189**	**39.53**	**0.005**	**0.025**	**0.34**	**0.15–0.75**

N: number of individuals, n: number of alleles (2n), %: allele frequency, p: Fisher’s exact test (p ≤ 0.05), pc: corrected p value, OR: odds ratio, CI: confidence interval, boldface: significant association after Bonferroni´s correction.

In the BT group, the analysis showed positive associations with HLA-A*33 (11.43% *vs.* 5.23%; p = 0.056, OR = 2.34, 95% CI = 1.01–5.41, pc = 1.12), HLA-B*53 (8.57% *vs.* 2.93%; p = 0.031, OR = 3.11, 95% CI = 1.15–8.37, pc = 0.992), HLA-C*16 (12.86% *vs.* 5.44%; p = 0.031, OR = 2.56, 95% CI = 1.15–5.73, pc = 0.434), and HLA-DRB1*03 (28.57% *vs.* 17.78%; p = 0.049, OR = 1.85, 95% CI = 1.05–3.27, pc = 0.637) and negative associations with HLA-B*40 (2.86% *vs.* 10.46%; p = 0.047, OR = 0.25, 95% CI = 0.06–1.06, pc = 1.50), HLA-B*49 (0.0% *vs.* 6.07%; p = 0.039, OR = 0.11, 95% CI = 0.006–1.79, pc = 1.24), and HLA-C*05 (4.28% *vs*. 14.02%; p = 0.020, OR = 0.27, 95% CI = 0.08–0.89, pc = 0.28). These results did not remain significant after p value correction (Table 2).

HLA allele frequencies that differed significantly between BL patients and healthy controls are shown in Table 2. HLA-B*53 was more common in BL patients (9.09% *vs.* 2.93%; p = 0.056, OR = 3.31, 95% CI = 1.04–10.54, pc = 1.792), whereas HLA-DRB1*07 (11.36% *vs* 26.77%; p = 0.028, OR = 0.35, 95% CI = 0.13–0.91, pc = 0.364) and HLA-DQB1*02 (18.18% *vs.* 39.53%; p = 0.005, 95% CI = 0.15–0.75, pc = 0.025) were more frequent in the controls. After p value correction, only the negative association between HLA-DQB1*02 and BL leprosy remained significant, which suggests that it may have a protective effect against BL leprosy.

### HLA alleles and leprosy reactions

The frequency of the HLA alleles in patients with leprosy reactions was analyzed only in the group of patients who had reversal reactions because only a small number of patients had ENL. In this analysis, patients who had reversal reactions were compared to patients without leprosy reactions, and the results are shown in Table 3. HLA-B*15 (28.72% *vs.* 12.76%; p = 0.011, OR = 2.75, 95% CI = 1.30–5.85, pc = 0.352) was positively correlated with reversal reactions; however, this correlation did not remain significant after correction.

**Table 3:** **HLA alleles with statistically significant differences in frequency between patients with reversal reactions and those with no reaction**

**HLA allele**	**Reversal reaction (N = 94)**		**No reaction (N = 94)**		**p**	**pc**	**OR**	**IC**
	**n**	**%**	**n**	**%**				
B*15	27	28.72	12	12.76	0.011	0.672	2.75	1.30–5.85

N: number of individuals, n: number of alleles (2n), %: allele frequency, p: Fisher’s exact test (p ≤ 0.05), pc: corrected p value, OR: odds ratio, CI: confidence interval.

### HLA haplotypes and B leprosy

Using the computational model in Arlequin 3.0, we obtained the expected frequencies of the HLA haplotypes in patients with B leprosy and healthy controls. In the B leprosy patients, it was possible to estimate 287 different haplotypes, and in the controls, it was possible to estimate 660 haplotypes. Table 4 shows only the most frequent haplotypes in the leprosy patients (greater than 1%) compared to the corresponding haplotypes in the controls. Due to the small number of patients in the study, we only analyzed B leprosy. The frequency of haplotypes A*02-B*07-C*07-DRB1*15-DQB1*06 and A*02-B*40-C*03-DRB1*13-DQB1*06 in B leprosy patients differed significantly from that in the controls (0.029703 *vs.* 0.010460, p = 0.015 and 0.017327 *vs.* 0.001046, p = 0.0011, respectively), suggesting an association between these haplotypes and susceptibility to this form of leprosy.

**Table 4:** **The HLA haplotypes in 202 B leprosy patients with frequencies above 1% and the frequencies in 478 controls**

**HLA haplotype**	**B leprosy Hf**	**Control Hf**	**p**
A*02-B*07-C*07-DRB1*15-DQB1*06	**0.029703**	**0.010460**	**0.015**
A*01-B*08-C*07-DRB1*03-DQB1*02	0.027228	0.018828	1.000
A*02-B*40-C*03-DRB1*13-DQB1*06	**0.017327**	**0.001046**	**0.0011**
A*03-B*14-C*08-DRB1*01-DQB1*05	0.014851	0.006276	0.197
A*02-B*15-C*03-DRB1*04-DQB1*03	0.014851	0.007101	1.000
A*24-B*07-C*07-DRB1*15-DQB1*06	0.012376	0.002092	0.369
A*02-B*51-C*15-DRB1*13-DQB1*06	0.012376	0.005230	0.317

Hf: haplotype frequency, p: Fisher’s exact test (p ≤ 0.05), boldface: significant association.

We performed a comparative analysis of the alleles that were previously reported to be associated with the polar forms of leprosy (HLA-DRB1*15, HLA-DRB1*16, and HLA-DRB1*03 for TT and HLA-DQB1*05 and HLA-DQB1*06 for LL) by direct counting. These alleles were also present in B leprosy patients. The results of the analysis are shown in Table 5. When analyzed separately, only the HLA-DRB1*16/DQB1*05 haplotype showed a statistically significant association (0.0891 *vs.* 0.0439; p = 0.030). Although HLA-DRB1*16/DQB1*06 was significant, it was only found in the controls (0.0000 *vs.* 0.0420; p = 0.029). When the analysis was extended to all possible haplotypes, this association was most significant, with a p value of 0.0055.

**Table 5:** The frequencies of HLA-DRB1*15, HLA-DRB1*16, HLA-DRB1*03, HLA-DQB1*05, and HLA-DQB1*06 in 202 patients with B leprosy and 478 controls

**Haplotype**	**Patients (N = 202)**		**Controls (N = 478)**		**p**
	**N**	**AF**	**n**	**AF**	
DRB1*15/DQB1*05 (#)	3	0.0148	3	0.0063	0.369
DRB1*15/DQB1*06	46	0.2277	82	0.1715	0.106
DRB1*16/DQB1*05	**18**	**0.0891**	**21**	**0.0439**	**0.030**
DRB1*16/DQB1*06 (#)	**0**	**0.0000**	**2**	**0.0420**	**0.029**
DRB1*03/DQB1*05 (#)	0	0.0000	0	0.0000	1.000
DRB1*03/DQB1*06 (#)	0	0.0000	1	0.0021	1,000
**Total**	**67**	**0.3317**	**109**	**22.80**	**0.0055**
**Others alleles**	135	0.6683	369	77.19	

N: number of individuals, n: number of alleles (2n), AF: allele frequency, p: Fisher’s exact test (p ≤ 0.05), (#) haplotypes that are not in linkage disequilibrium, boldface: significant association.

Table 6 summarizes the major findings of our B leprosy susceptibility and protection HLA allele association analysis along with a comparison to data from previous studies of different populations.

**Table 6:** **Major findings in this study compared to previous data**

**HLA association**	**B**	**BB**	**BT**	**BL**	**Reaction**	**Associated phenotype**	**Population**	**Reference**
A*33	-	-	Susc	-	-	Susc (L. *per se*)	Korea	22
B*15	-	-	-	-	Susc	-	-	-
B*58	-	Susc	-	-	-	-	-	-
B*40	-	-	Prot	-	-	-	-	-
B*49	Prot	Prot	Prot	-	-	Prot (L. *per se*)	Turkey	35
B*53	-	-	Susc	Susc	-	-	-	-
C*05	Prot	Prot	Prot	-	-	Prot (L. *per se)*	Brazil	37
C*12	-	Susc	-	-	-	Susc (L. p*er se*)	Brazil	11
C*16	-	-	Susc	-	-	Prot (L. *per se)*	Brazil	11
DRB1*03	-	-	Susc	Prot	-	TT	Different populations	16,34
DRB1*07	Prot	-	-	Prot	-	Prot (MB)	India	18
DQB1*02	-	-	-	Prot	-	Prot (MB)	India and Argentina	18,38
DQB1*04	-	-	-	-	Susc	-	-	-

B: borderline, BB: mid-borderline, BT: borderline tuberculoid, BL: borderline lepromatous, TT: tuberculoid leprosy; Susc: associated with susceptibility; Prot: protective association; L. *per se*: leprosy *per se*, MB: multibacillary leprosy, PB: paucibacillary leprosy; (−): no description in the literature.

## Discussion

Studies on the involvement of the HLA complex in leprosy have consistently shown that this major histocompatibility region contains the major genes associated with susceptibility to the disease [39]. Many HLA class II molecules have been consistently found to be associated with different clinical phenotypes and disease *per se*. Studies of HLA class I initially did not consider the possibility that presentation by T-CD8+ lymphocytes could be important in leprosy. However, the positive association between HLA class I and leprosy reflects the role of these cells in the production of IFN-γ in response to *M. leprae* antigens [39].

Previous studies of different clinical forms of leprosy mainly included just the polar forms LL and TT. The consensus in the literature is that HLA-DR2 (subtypes HLA-DRB1*15 and HLA-DRB1*16) and HLA-DR3 are associated with the TT form [19-23,27,40] and HLA-DQB1*01 (subtypes HLA-DQB1*05 and HLA-DQB1*06) is associated with the LL form [25,26]. No previous studies included only the intermediate clinical forms of the disease (BT, BB, and BL). Therefore, the current study was proposed.

The results showed decreased frequencies of HLA-C*05 and HLA-DRB1*07 in B leprosy patients when compared to the healthy control individuals, suggesting that they are associated with protection against B leprosy. Analysis of HLA-B*49 showed a pc value that was nearly significant, suggesting a possible protective association. HLA-B*49 was found to be significantly associated with protection against leprosy *per se* in a Turkish population [41].

In a Brazilian population, HLA-C*05 is 1 of the 5 most frequent alleles at this locus (approximately 14.0% in our control group), and HLA-DRB1*07 is the most frequent (26.77% in our control group) [42]. In B leprosy patients, we observed decreased frequencies for these 2 alleles (5.94% for HLA-C*05 and 16.34% for HLA-DRB1*07). The protective effect of HLA-C*05 against in leprosy *per se* was recently described in a population in Rio de Janeiro, Brazil. However, in this study, the patients were not divided into groups according to their clinical forms; therefore, it was not possible to verify whether this allele was associated with B leprosy [43]. Rani *et al*. [24] reported a decreased frequency of HLA-DRB1*0701 in LL and BL patients compared to TT patients and healthy controls in a population from Northern India, and they assigned this allele a protective effect against multibacillary (MB) forms. HLA-DRB1*1501 and HLA-DQB1*0602 were associated with susceptibility in MB patients in Japan, and no allele was associated with protection in this population [34]. Silva *et al*. [32] reported that the HLA-DRB1*1601 allele was associated with susceptibility to B leprosy in a Brazilian population. In our study, no allele was associated with susceptibility.

Significant susceptibility and protective associations were observed in BB, BL, and BT, although the corrected p was higher than the significance level (5%). To confirm the associations in these clinical forms, the groups were compared 2 ways: BB, BL, and BT were compared with the healthy control group, and then each group (i.e., BB, BL, or BT) was compared to patients without the clinical form analyzed (see Additional file 2).

In the BB group, a protective association was observed for HLA-B*49 and HLA-C*05 when compared to the healthy controls. These same alleles were also found in B leprosy. In both analyses (compared to healthy controls and patient controls), HLA-B*58 and HLA-C*12 were associated with susceptibility to the BB form. HLA-C*12 was previously shown to be associated with susceptibility to leprosy *per se* in a population in Southeast Brazil [17]; however, no study has shown an association between HLA-B*58 and leprosy. Although our statistical analyses did not support susceptibility (*p*c ≥ 0.05), the finding of HLA-B*58 in our patient group suggests that this allele is a specific marker for clinical BB leprosy in our region, as the frequency of this allele in the general population is approximately 5.0%, whereas in BB patients, the frequency was 11.36% [42].

In BL patients, HLA-B*53 was associated with susceptibility when compared to the healthy controls; however, similar results were not observed when compared to the patient controls. The protective associations of HLA-DRB1*07 and HLA-DRB1*03 differed in the analyses with different control groups; HLA-DRB1*07 was significant when compared to the healthy controls, whereas HLA-DRB1*03 was significant when compared to the patient controls. Unlike the associations described above, HLA-DQB1*02 was associated with protection in BL patients when compared to both control groups, and a statistically significant association was maintained after Bonferroni correction.

Rani *et al*. [24] also reported a protective association for HLA-DQB1*02 as well as HLA-DRB1*07 in MB leprosy patients in India. Motta *et al.* [44] studied 89 leprosy patients (70 with MB and 19 with paucibacillary [PB]) and 112 healthy controls in Argentina (province of Chaco), and confirmed HLA-DQB1*02 as a protective factor against the MB form. In this study, they also showed that HLA-DRB1*04 was associated with protection against the PB form. However, in our study, this association was not found in B leprosy patients, suggesting that HLA-DQB1*02 is specific to the BL form or MB patients.

In the BT group, a protective association was found for HLA-B*40 when compared to both control groups, whereas HLA-B*49 and HLA-C*05 were only found to be protective when compared to the healthy control group. HLA-B*49 and HLA-C*05 were also associated with protection against B leprosy. HLA-B*53 and HLA-C*16 were associated with susceptibility only when compared to the healthy controls, whereas HLA-A*33 and HLA-DRB1*03 were associated with susceptibility when compared to both control groups. Kim *et al*. [28] showed that HLA-A*33 was shown associated with susceptibility to leprosy *per se* in Korea.

Studies [22,40,45] of different populations have demonstrated an association between HLA-DRB1*03 and predisposition to TT. In our study, the association of this allele with BT suggests that it has an effect on susceptibility to this clinical form, as this same allele was found to be associated with protection against the BL form.

These results suggest that HLA-DRB1*03 is associated with polarization to the B form and manifestation of this clinical form. Until now, this is a controversial point in the diagnosis of leprosy [3,4]. To better understand and confirm this result, the frequency of this allele in the polar forms of leprosy (LL and TT) should be determined, and these results should be replicated in other independent populations.

Due the small number of patients with ENL, they could not be studied. Therefore, only patients with reversal reactions were included, and a significant association with HLA-B*15 was found. Although this result lost significance after statistical correction, this finding must be considered, as the frequency of this allele differs considerably between patients with reversal reactions and non-reactional patients. In patients with reversal reactions, the frequency of HLA-B*15 was approximately 29.0%, whereas in the non-reaction group, the frequency was 13.0%.

In the literature, there are few studies describing the association between HLA and leprosy reactions. Ottenhoff found an association between HLA-DR3 and reactions in an Ethiopian population; however, this study involved a specific group of patients, Mitsuda-positive BT leprosy patients with reversal reactions. HLA-DR3 was shown to be involved in the acquisition of cellular immunity during the reactional state, but not in predisposition to this form of the disease [46]. In our study, HLA-DR3 was not associated with reactions. In addition, a negative association with BL was observed, which reinforces the hypothesis that HLA-DR3 is associated with BT leprosy and polarization in B leprosy.

The haplotype analysis showed that haplotypes A*02-B*07-C*07-DRB1*15-DQB1*06 and A*02-B*40-C*03-DRB1*13-DQB1*06 were more frequent in B patients, and the difference between the frequencies in B leprosy patients and the controls was significant, suggesting a possible role in susceptibility to B leprosy. In Indian populations [15,16], MB leprosy was associated with haplotypes A*1102-B*4006-Cw*1502 and A*0203-B*4016-Cw*0703. Although the haplotypes are different, the B*40 allele found in these Indian populations was also detected in our population. However, it is not possible to say whether this allele genotype is the same as that observed in our population. In the Rio de Janeiro population, the haplotypes associated with susceptibility to leprosy *per se* were A*02-B*35-C*04, A*02-B*15-C*03, A*30-B*42-C*17, A*03-B*35-C*04, A*26-B*38-C*12, and A*24-B*15-C*03, which differ from those found in our population [43].

Interestingly, the class II alleles present in both haplotypes found in our population, were previously shown to be associated with the clinical forms TT (DRB1*15) and LL (DQB1*06). This observation caused us to examine these class II alleles in B patients in greater detail. When analyzed independently, the results showed that haplotype HLA-DRB1*16/DQB1*05 was associated with susceptibility.

Analysis of all possible haplotypes together (DRB1*15/DQB1*05, DRB1*15/DQB1*06, DRB1*16/DQB1*05, DRB1*16/DQB1*06, DRB1*03/DQB1*05, and DRB1*03/DQB1*06) showed a greater association with susceptibility. Thus, we hypothesized that the presence of alleles associated with the polar clinical forms LL and TT may induce the manifestation of B leprosy by triggering the intermediate immune response that is characteristic of this form of the disease.

## Conclusions

The results of the present study showed the involvement of HLA class I and class II alleles in B leprosy and reversal reaction episodes and suggested a role for HLA molecules in polarization in this group of patients. Our findings provide a greater understanding of the immune responses exhibited by patients with B leprosy. In the future, similar studies of different populations are needed to confirm these findings.

## Additional files

Additional file 1:
**1.1 HLA-A*, B*, C*, DRB1* and DQB1* alleles frequencies in 202 patients with B leprosy and 478 healthy control.** **1.2.** HLA-A*, B*, C*, DRB1* and DQB1* alleles frequencies in 88 BB leprosy and 478 healthy controls. **1.3.** HLA-A*, B*, C*, DRB1* and DQB1* alleles frequencies in 70 BT leprosy and 478 healthy controls. **1.4.** HLA-A*, B*, C*, DRB1* and DQB1* alleles frequencies in 44 BL leprosy and 478 healthy controls. **1.5.** HLA-A*, B*, C*, DRB1* and DQB1* alleles frequencies in 94 leprosy patients with reversal reaction and 94 no reaction leprosy. Additional file 2:
**2.1 HLA-A*, B*, C*, DRB1* and DQB1* alleles frequencies in 70 BT leprosy and 132 BB and BL leprosy patients controls.** **2.2.** HLA-A*, B*, C*, DRB1* and DQB1* alleles frequencies in 88 BB leprosy and 114 BT and BL leprosy patients controls. **2.3.** HLA-A*, B*, C*, DRB1* and DQB1* alleles frequencies in 44 BL leprosy and 158 BT and BB leprosy patients controls.

## Abbreviations

*M. leprae*: *Mycobacterium leprae*
HLA: Human leukocyte antigen
TCR: T-cell receptor
TT: Tuberculoid leprosy
LL: Lepromatous leprosy
B: Borderline leprosy
BT: Borderline-tuberculoid
BB: Borderline-borderline
BL: Borderline-lepromatous
ILSL: Lauro de Souza Lima Institute
PCR-SSO: Polymerase chain reaction-sequence specific oligonucleotide
EM: Expectation maximization
MB: Multibacillary leprosy
PB: Paucibacillary leprosy
