# Supplementary material for: Human leukocyte antigen class I and class II alleles are associated with susceptibility and resistance in borderline leprosy patients from Southeast Brazil
Source: BMC Infect Dis. 2015 Jan 21;15:22. doi: 10.1186/s12879-015-0751-0 (PMC4307149; doi:10.1186/s12879-015-0751-0)
Supplement: Additional file 1: — 1.1 HLA-A*, B*, C*, DRB1* and DQB1* alleles frequencies in 202 patients with B leprosy and 478 healthy control. 1.2. HLA-A*, B*, C*, DRB1* and DQB1* alleles frequencies in 88 BB leprosy and 478 healthy controls. 1.3. HLA-A*, B*, C*, DRB1* and DQB1* alleles frequencies in 70 BT leprosy and 478 healthy controls. 1.4. HLA-A*, B*, C*, DRB1* and DQB1* alleles frequencies in 44 BL leprosy and 478 healthy controls. 1.5. HLA-A*, B*, C*, DRB1* and DQB1* alleles frequencies in 94 leprosy patients with reversal reaction and 94 no reaction leprosy. [file 12879_2015_751_MOESM1_ESM.pdf]

Additional file 1.1. HLA-A*, B*, C*, DRB1* and DQB1* alleles frequencies in 202 patients with B leprosy and 478 healthy controls.

HLA allele	B leprosy (N=202)		Controls (N= 478)		p	HLA allele	B leprosy (N=202)		Controls (N= 478)		p
	n	Fa (%)	n	Fa (%)			n	Fa (%)	n	Fa (%)	
**A*01**	32	15.84	82	17.15	0.736	**A*31**	13	6.43	48	10.04	0.144
**A*02**	107	53.00	236	49.37	0.402	**A*32**	11	5.44	34	7.11	0.501
**A*03**	50	24.75	95	19.87	0.182	**A*33**	12	5.94	25	5.23	0.713
**A*11**	22	10.89	56	11.71	0.794	**A*34**	02	0.99	11	2.30	0.363
**A*23**	14	6.93	47	9.83	0.244	**A*36**	02	0.99	06	1.25	1.000
**A*24**	40	19.80	101	21.13	0.756	**A*66**	02	0.99	05	1.05	1.000
**A*25**	03	1.48	13	2.72	0.416	**A*68**	25	12.38	51	10.67	0.508
**A*26**	20	9.90	38	7.95	0.452	**A*69**	00	0.00	03	0.63	0.558
**A*29**	16	7.92	48	10.04	0.472	**A*74**	05	2.47	04	0.84	0.134
**A*30**	27	13.37	52	10.88	0.361	**A*80**	01	0.49	01	0.21	0.506
**B*07**	38	18.81	61	12.78	0.043	**B*47**	02	0.99	00	0.00	0.087
**B*08**	25	12.38	49	10.27	0.420	**B*48**	01	0.49	03	0.62	1.000
**B*13**	08	3.96	22	4.61	0.839	**B*49**	02	0.99	29	6.07	0.002
**B*14**	20	9.90	47	9.85	1.000	**B*50**	04	1.98	30	6.28	0.019
**B*15**	41	20.30	80	16.77	0.273	**B*51**	35	17.33	86	18.02	0.912
**B*18**	25	12.38	64	13.41	0.803	**B*52**	07	3.46	19	3.98	0.830
**B*27**	04	1.98	20	4.19	0.178	**B*53**	13	6.43	14	2.93	0.050
**B*35**	44	21.78	103	21.55	1.000	**B*54**	00	0.00	01	0.20	1.000
**B*37**	04	1.98	13	2.72	0.789	**B*55**	03	1.48	14	2.93	0.420
**B*38**	09	4.45	21	4.40	1.000	**B*56**	01	0.49	04	0.83	1.000
**B*39**	15	7.42	30	6.28	0.613	**B*57**	10	4.95	25	5.24	1.000
**B*40**	23	11.39	50	10.46	0.786	**B*58**	12	5.94	24	5.03	0.707
**B*41**	02	0.99	04	0.83	1.000	**B*59**	00	0.00	01	0.20	1.000
**B*42**	06	2.97	19	3.98	0.657	**B*73**	00	0.00	01	0.20	1.000
**B*44**	35	17.33	98	20.54	0.397	**B*78**	00	0.00	01	0.20	1.000
**B*45**	10	4.95	18	3.77	0.527	**B*81**	05	2.47	05	1.05	0.172
**C*01**	08	3.96	34	7.11	0.161	**C*08**	25	12.38	47	9.83	0.340
**C*02**	18	8.91	63	13.18	0.122	**C*12**	31	15.35	64	13.39	0.545
**C*03**	53	26.24	104	21.75	0.232	**C*14**	08	3.96	18	3.76	1.000
**C*04**	61	30.20	144	30.12	1.000	**C*15**	20	9.90	50	10.46	0.890
**C*05**	12	5.94	67	14.02	0.002	**C*16**	21	10.4	26	5.44	0.030
**C*06**	36	17.82	96	20.08	0.525	**C*17**	08	3.96	23	4.81	0.692
**C*07**	99	49.00	216	45.19	0.400	**C*18**	04	1.98	04	0.84	0.246
**DRB1*01**	38	18.81	94	19.66	0.832	**DRB1*11**	55	27.23	108	22.59	0.202
**DRB1*03**	41	20.30	85	17.78	0.450	**DRB1*12**	04	1.98	15	3.13	0.610
**DRB1*04**	38	18.81	87	18.20	0.913	**DRB1*13**	64	31.68	155	32.42	0.928
**DRB1*07**	33	16.34	128	26.77	0.003	**DRB1*14**	19	9.40	56	11.71	0.423
**DRB1*08**	20	9.90	36	7.53	0.359	**DRB1*15**	55	27.23	111	23.22	0.283
**DRB1*09**	06	2.97	20	4.18	0.519	**DRB1*16**	22	10.9	36	7.53	0.175
**DRB1*10**	09	4.45	25	5.23	0.847						
**DQB1*02**	67	33.17	189	39.53	0.120	**DQB1*05**	93	46.04	192	40.16	0.173
**DQB1*03**	119	58.91	298	62.34	0.438	**DQB1*06**	106	52.47	224	46.86	0.207
**DQB1*04**	19	9.40	53	11.08	0.586						

N: number of individuals, n: number of alleles (2n), %: allele frequency, p: Fisher's exact test ($p \le 0.05$).

Additional file 1.2. HLA-A*, B*, C*, DRB1* and DQB1* alleles frequencies in 88 BB leprosy and 478 healthy controls.

HLA allele	BB leprosy (N=88)		Controls (N=478)		p	HLA allele	BB leprosy (N=88)		Controls (N=478)		p
	n	Fa (%)	n	Fa (%)			n	Fa (%)	n	Fa (%)	
**A*01**	17	19.31	82	17.15	0.647	**A*31**	07	7.95	48	10.04	0.695
**A*02**	44	50.00	236	49.37	1.000	**A*32**	04	4.54	34	7.11	0.490
**A*03**	21	23.86	95	19.87	0.391	**A*33**	03	3.41	25	5.23	0.600
**A*11**	14	15.90	56	11.71	0.290	**A*34**	02	2.27	11	2.30	1.000
**A*23**	04	4.54	47	9.83	0.153	**A*36**	00	0.00	06	1.25	0.597
**A*24**	19	21.59	101	21.13	0.888	**A*66**	00	0.00	05	1.05	1.000
**A*25**	01	1.14	13	2.72	0.707	**A*68**	13	14.77	51	10.67	0.272
**A*26**	10	11.36	38	7.95	0.298	**A*69**	00	0.00	03	0.63	1.000
**A*29**	05	5.68	48	10.04	0.235	**A*74**	01	1.14	04	0.84	0.571
**A*30**	11	12.50	52	10.88	0.711	**A*80**	00	0.00	01	0.21	1.000
**B*07**	13	14.77	61	12.78	0.606	**B*47**	01	1.14	00	0.00	0.155
**B*08**	12	13.64	49	10.27	0.350	**B*48**	00	0.00	03	0.62	1.000
**B*13**	02	2.27	22	4.61	0.561	**B*49**	00	0.00	29	6.07	0.014
**B*14**	10	11.36	47	9.85	0.699	**B*50**	01	1.14	30	6.28	0.069
**B*15**	13	14.77	80	16.77	0.754	**B*51**	12	13.64	86	18.02	0.361
**B*18**	10	11.36	64	13.41	0.731	**B*52**	05	5.68	19	3.98	0.400
**B*27**	04	4.54	20	4.19	0.778	**B*53**	04	4.54	14	2.93	0.503
**B*35**	21	23.86	103	21.55	0.674	**B*54**	00	0.00	01	0.20	1.000
**B*37**	03	3.41	13	2.72	0.724	**B*55**	01	1.14	14	2.93	0.486
**B*38**	05	5.68	21	4.40	0.580	**B*56**	01	1.14	04	0.83	0.571
**B*39**	04	4.54	30	6.28	0.633	**B*57**	05	5.68	25	5.24	0.798
**B*40**	13	14.77	50	10.46	0.267	**B*58**	10	11.36	24	5.03	0.028
**B*41**	02	2.27	04	0.83	0.236	**B*59**	00	0.00	01	0.20	1.000
**B*42**	03	3.41	19	3.98	1.000	**B*73**	00	0.00	01	0.20	1.000
**B*44**	16	18.18	98	20.54	0.667	**B*78**	00	0.00	01	0.20	1.000
**B*45**	04	4.54	18	3.77	0.762	**B*81**	01	1.14	05	1.05	1.000
**C*01**	05	5.68	34	7.11	0.819	**C*08**	12	13.64	47	9.83	0.340
**C*02**	07	7.95	63	13.18	0.217	**C*12**	19	21.59	64	13.39	0.050
**C*03**	20	22.73	104	21.75	0.888	**C*14**	00	0.00	18	3.76	0.091
**C*04**	25	28.41	144	30.12	0.800	**C*15**	08	9.09	50	10.46	0.848
**C*05**	05	5.68	67	14.02	0.035	**C*16**	07	7.95	26	5.44	0.328
**C*06**	21	23.86	96	20.08	0.473	**C*17**	04	4.54	23	4.81	1.000
**C*07**	41	46.59	216	45.19	0.816	**C*18**	02	2.27	04	0.84	0.236
**DRB1*01**	14	15.91	94	19.66	0.463	**DRB1*11**	26	29.54	108	22.59	0.172
**DRB1*03**	17	19.32	85	17.78	0.762	**DRB1*12**	01	1.14	15	3.13	0.487
**DRB1*04**	18	20.45	87	18.20	0.769	**DRB1*13**	25	28.41	155	32.42	0.533
**DRB1*07**	16	18.18	128	26.77	0.109	**DRB1*14**	10	11.36	56	11.71	1.000
**DRB1*08**	06	6.82	36	7.53	1.000	**DRB1*15**	26	29.54	111	23.22	0.223
**DRB1*09**	01	1.14	20	4.18	0.226	**DRB1*16**	10	11.36	36	7.53	0.285
**DRB1*10**	06	6.82	25	5.23	0.608						
**DQB1*02**	29	32.95	189	39.53	0.283	**DQB1*05**	41	46.59	192	40.16	0.289
**DQB1*03**	54	61.36	298	62.34	0.904	**DQB1*06**	45	51.14	224	46.86	0.487
**DQB1*04**	07	7.95	53	11.08	0.454						

N: number of individuals, n: number of alleles (2n), %: allele frequency, p: Fisher's exact test ($p \leq 0.05$).

Additional file 1.3. HLA-A*, B*, C*, DRB1* and DQB1* alleles frequencies in 70 BT leprosy and 478 healthy controls.

HLA allele	BT leprosy (N=70)		Controls (N=478)		p	HLA allele	BT leprosy (N=70)		Controls (N=478)		p
	n	Fa (%)	n	Fa (%)			n	Fa (%)	n	Fa (%)	
**A*01**	13	18.57	82	17.15	0.737	**A*31**	04	5.71	48	10.04	0.380
**A*02**	37	52.86	236	49.37	0.610	**A*32**	03	4.28	34	7.11	0.607
**A*03**	19	27.14	95	19.87	0.159	**A*33**	08	11.43	25	5.23	0.056
**A*11**	05	7.14	56	11.71	0.313	**A*34**	00	0.00	11	2.30	0.374
**A*23**	04	5.71	47	9.83	0.377	**A*36**	01	1.43	06	1.25	1.000
**A*24**	13	18.57	101	21.13	0.752	**A*66**	02	2.86	05	1.05	0.221
**A*25**	02	2.86	13	2.72	1.000	**A*68**	08	11.43	51	10.67	0.836
**A*26**	05	7.14	38	7.95	1.000	**A*69**	00	0.00	03	0.63	1.000
**A*29**	04	5.71	48	10.04	0.380	**A*74**	02	2.86	04	0.84	0.171
**A*30**	10	14.28	52	10.88	0.418	**A*80**	00	0.00	01	0.21	1.000
**B*07**	15	21.43	61	12.78	0.062	**B*47**	01	1.43	00	0.00	0.127
**B*08**	10	14.28	49	10.27	0.304	**B*48**	01	1.43	03	0.62	0.422
**B*13**	03	4.28	22	4.61	1.000	**B*49**	00	0.00	29	6.07	0.039
**B*14**	08	11.43	47	9.85	0.670	**B*50**	01	1.43	30	6.28	0.160
**B*15**	16	22.85	80	16.77	0.237	**B*51**	16	22.85	86	18.02	0.326
**B*18**	11	15.71	64	13.41	0.578	**B*52**	02	2.86	19	3.98	1.000
**B*27**	00	0.00	20	4.19	0.093	**B*53**	06	8.57	14	2.93	0.031
**B*35**	14	20.00	103	21.55	0.876	**B*54**	00	0.00	01	0.20	1.000
**B*37**	02	2.86	13	2.72	1.000	**B*55**	02	2.86	14	2.93	1.000
**B*38**	02	2.86	21	4.4	0.754	**B*56**	00	0.00	04	0.83	1.000
**B*39**	06	8.57	30	6.28	0.441	**B*57**	03	4.28	25	5.24	1.000
**B*40**	02	2.86	50	10.46	0.047	**B*58**	02	2.86	24	5.03	0.559
**B*41**	00	0.00	04	0.83	1.000	**B*59**	00	0.00	01	0.20	1.000
**B*42**	03	4.28	19	3.98	0.752	**B*73**	00	0.00	01	0.20	1.000
**B*44**	09	12.86	98	20.54	0.149	**B*78**	00	0.00	01	0.20	1.000
**B*45**	02	2.86	18	3.77	1.000	**B*81**	03	4.28	05	1.05	0.069
**C*01**	02	2.86	34	7.11	0.297	**C*08**	09	12.85	47	9.83	0.403
**C*02**	04	5.71	63	13.18	0.080	**C*12**	08	11.43	64	13.39	0.849
**C*03**	17	24.28	104	21.75	0.644	**C*14**	04	5.71	18	3.76	0.508
**C*04**	24	34.28	144	30.12	0.489	**C*15**	10	14.28	50	10.46	0.311
**C*05**	03	4.28	67	14.02	0.020	**C*16**	09	12.86	26	5.44	0.031
**C*06**	09	12.85	96	20.08	0.192	**C*17**	04	5.71	23	4.81	0.765
**C*07**	35	50.00	216	45.19	0.521	**C*18**	02	2.86	04	0.84	0.171
**DRB1*01**	11	15.71	94	19.66	0.516	**DRB1*11**	15	21.43	108	22.59	0.879
**DRB1*03**	20	28.57	85	17.78	0.049	**DRB1*12**	02	2.86	15	3.13	1.000
**DRB1*04**	11	15.71	87	18.20	0.738	**DRB1*13**	26	37.14	155	32.42	0.496
**DRB1*07**	12	17.14	128	26.77	0.105	**DRB1*14**	05	7.14	56	11.71	0.313
**DRB1*08**	07	10.00	36	7.53	0.474	**DRB1*15**	17	24.28	111	23.22	0.879
**DRB1*09**	04	5.71	20	4.18	0.531	**DRB1*16**	09	12.86	36	7.53	0.157
**DRB1*10**	01	1.43	25	5.23	0.231						
**DQB1*02**	30	42.85	189	39.53	0.603	**DQB1*05**	30	42.85	192	40.16	0.696
**DQB1*03**	36	51.43	298	62.34	0.088	**DQB1*06**	37	52.85	224	46.86	0.371
**DQB1*04**	07	10.00	53	11.08	1.000						

N: number of individuals, n: number of alleles (2n), %: allele frequency, p: Fisher's exact test ($p \leq 0.05$).

Additional file 1.4. HLA-A*, B*, C*, DRB1* and DQB1* alleles frequencies in 44 BL leprosy and 478 healthy controls.

HLA allele	BL leprosy (N=44)		Controls (N=478)		p	HLA allele	BL leprosy (N=44)		Controls (N=478)		p
	n	Fa (%)	n	Fa (%)			n	Fa (%)	n	Fa (%)	
**A*01**	03	6.82	82	17.15	0.087	**A*31**	02	4.54	48	10.04	0.417
**A*02**	26	59.09	236	49.37	0.270	**A*32**	04	9.09	34	7.11	0.549
**A*03**	10	22.73	95	19.87	0.694	**A*33**	01	2.27	25	5.23	0.714
**A*11**	03	6.82	56	11.71	0.456	**A*34**	00	0.00	11	2.30	0.611
**A*23**	06	13.64	47	9.83	0.431	**A*36**	01	2.27	06	1.25	0.462
**A*24**	08	18.18	101	21.13	0.846	**A*66**	00	0.00	05	1.05	1.000
**A*25**	00	0.00	13	2.72	0.614	**A*68**	04	9.09	51	10.67	1.000
**A*26**	04	9.09	38	7.95	0.771	**A*69**	00	0.00	03	0.63	1.000
**A*29**	07	15.91	48	10.04	0.207	**A*74**	02	4.54	04	0.84	0.083
**A*30**	06	13.64	52	10.88	0.614	**A*80**	01	2.27	01	0.21	0.161
**B*07**	10	22.73	61	12.78	0.103	**B*47**	00	0.00	00	0.00	1.000
**B*08**	03	6.82	49	10.27	0.605	**B*48**	00	0.00	03	0.62	1.000
**B*13**	02	4.54	22	4.61	1.000	**B*49**	02	4.54	29	6.07	1.000
**B*14**	02	4.54	47	9.85	0.414	**B*50**	02	4.54	30	6.28	1.000
**B*15**	11	25.00	80	16.77	0.209	**B*51**	07	15.91	86	18.02	0.839
**B*18**	04	9.09	64	13.41	0.638	**B*52**	00	0.00	19	3.98	0.391
**B*27**	00	0.00	20	4.19	0.399	**B*53**	04	9.09	14	2.93	0.056
**B*35**	09	20.45	103	21.55	1.000	**B*54**	00	0.00	01	0.20	1.000
**B*37**	00	0.00	13	2.72	0.614	**B*55**	00	0.00	14	2.93	0.620
**B*38**	02	4.54	21	4.40	1.000	**B*56**	00	0.00	04	0.83	1.000
**B*39**	05	11.36	30	6.28	0.202	**B*57**	02	4.54	25	5.24	1.000
**B*40**	08	18.18	50	10.46	0.131	**B*58**	00	0.00	24	5.03	0.249
**B*41**	00	0.00	04	0.83	1.000	**B*59**	00	0.00	01	0.20	1.000
**B*42**	00	0.00	19	3.98	0.391	**B*73**	00	0.00	01	0.20	1.000
**B*44**	10	22.73	98	20.54	0.699	**B*78**	00	0.00	01	0.20	1.000
**B*45**	04	9.09	18	3.77	0.104	**B*81**	01	2.27	05	1.05	0.412
**C*01**	01	2.27	34	7.11	0.345	**C*08**	04	9.09	47	9.83	1.000
**C*02**	07	15.91	63	13.18	0.642	**C*12**	04	9.09	64	13.39	0.638
**C*03**	15	34.09	104	21.75	0.088	**C*14**	04	9.09	18	3.76	0.104
**C*04**	13	29.54	144	30.12	1.000	**C*15**	02	4.54	50	10.46	0.294
**C*05**	04	9.09	67	14.02	0.491	**C*16**	05	11.36	26	5.44	0.169
**C*06**	06	13.64	96	20.08	0.425	**C*17**	00	0.00	23	4.81	0.245
**C*07**	23	52.27	216	45.19	0.429	**C*18**	00	0.00	04	0.84	1.000
**DRB1*01**	12	27.27	94	19.66	0.241	**DRB1*11**	14	31.82	108	22.59	0.191
**DRB1*03**	04	9.09	85	17.78	0.206	**DRB1*12**	01	2.27	15	3.13	1.000
**DRB1*04**	08	18.18	87	18.20	1.000	**DRB1*13**	14	31.82	155	32.42	1.000
**DRB1*07**	05	11.36	128	26.77	0.028	**DRB1*14**	04	9.09	56	11.71	0.805
**DRB1*08**	07	15.91	36	7.53	0.077	**DRB1*15**	13	29.54	111	23.22	0.356
**DRB1*09**	01	2.27	20	4.18	1.000	**DRB1*16**	03	6.82	36	7.53	1.000
**DRB1*10**	02	4.54	25	5.23	1.000						
**DQB1*02**	8	18.18	189	39.53	0.005	**DQB1*05**	21	47.73	192	40.16	0.340
**DQB1*03**	28	63.64	298	62.34	1.000	**DQB1*06**	26	59.09	224	46.86	0.155
**DQB1*04**	5	11.36	53	11.08	1.000						

N: number of individuals, n: number of alleles (2n), %: allele frequency, p: Fisher's exact test ($p \leq 0.05$).

Additional file 1.5. HLA-A*, B*, C*, DRB1* and DQB1* alleles frequencies in 94 leprosy patients with reversal reaction and 94 no reaction leprosy.

HLA allele	Reversal reaction (N=94)		No reaction (N=94)		p	HLA allele	Reversal reaction (N=94)		No reaction (N=94)		p
	n	Fa (%)	n	Fa (%)			n	Fa (%)	N	Fa (%)	
**A*01**	19	20.21	13	13.83	0.332	**A*31**	05	5.32	07	7.45	0.766
**A*02**	52	55.32	43	45.74	0.243	**A*32**	05	5.32	04	4.25	1.000
**A*03**	25	26.59	21	22.34	0.611	**A*33**	04	4.25	06	6.38	0.747
**A*11**	07	7.45	14	14.89	0.163	**A*34**	01	1.06	01	1.06	1.000
**A*23**	06	6.38	07	7.45	1.000	**A*36**	02	2.13	00	0.00	0.497
**A*24**	19	20.21	19	20.21	1.000	**A*66**	02	2.13	00	0.00	0.497
**A*25**	01	1.06	02	2.13	1.000	**A*68**	10	10.64	15	15.96	0.390
**A*26**	10	10.64	09	9.57	1.000	**A*69**	00	0.00	00	0.00	1.000
**A*29**	04	4.25	11	11.70	0.103	**A*74**	03	3.19	02	2.13	1.000
**A*30**	12	12.76	14	14.89	0.833	**A*80**	01	1.06	00	0.00	1.000
**B*07**	19	20.21	16	17.02	0.708	**B*47**	00	0.00	01	1.06	0.474
**B*08**	17	18.08	08	8.51	0.084	**B*48**	01	1.06	00	0.00	1.000
**B*13**	04	4.25	04	4.25	1.000	**B*49**	00	0.00	02	2.13	0.497
**B*14**	09	9.57	10	10.64	1.000	**B*50**	01	1.06	02	2.13	1.000
**B*15**	27	28.72	12	12.76	0.011	**B*51**	17	18.08	15	15.96	1.000
**B*18**	07	7.45	15	15.96	0.070	**B*52**	04	4.25	03	3.19	1.000
**B*27**	02	2.13	02	2.13	1.000	**B*53**	07	7.45	06	6.38	1.000
**B*35**	18	19.15	23	24.47	0.480	**B*54**	00	0.00	00	0.00	1.000
**B*37**	01	1.06	03	3.19	0.621	**B*55**	02	2.13	01	1.06	1.000
**B*38**	03	3.19	05	5.32	0.720	**B*56**	00	0.00	01	1.06	1.000
**B*39**	08	8.51	06	6.38	0.782	**B*57**	05	5.32	05	5.32	1.000
**B*40**	06	6.38	13	13.83	0.145	**B*58**	06	6.38	06	6.38	1.000
**B*41**	02	2.13	00	0.00	0.497	**B*59**	00	0.00	00	0.00	1.000
**B*42**	04	4.25	02	2.13	0.682	**B*73**	00	0.00	00	0.00	1.000
**B*44**	11	11.70	21	22.34	0.079	**B*78**	00	0.00	00	0.00	1.000
**B*45**	04	4.25	04	4.25	1.000	**B*81**	03	3.19	02	2.13	1.000
**C*01**	04	4.25	04	4.25	1.000	**C*08**	11	11.70	13	13.83	0.827
**C*02**	08	8.51	07	7.45	1.000	**C*12**	10	10.64	17	18.08	0.211
**C*03**	28	29.79	20	21.28	0.241	**C*14**	04	4.25	02	2.13	0.682
**C*04**	27	28.72	31	32.98	0.635	**C*15**	10	10.64	10	10.64	1.000
**C*05**	03	3.19	09	9.57	0.133	**C*16**	08	8.51	12	12.76	0.478
**C*06**	16	17.02	16	17.02	1.000	**C*17**	06	6.38	02	2.13	0.278
**C*07**	51	54.25	43	45.74	1.000	**C*18**	02	2.13	02	2.13	1.000
**DRB1*01**	16	17.02	17	18.08	1.000	**DRB1*11**	22	23.40	29	30.85	0.325
**DRB1*03**	25	26.59	15	15.96	0.108	**DRB1*12**	01	1.06	03	3.19	0.621
**DRB1*04**	19	20.21	18	19.15	1.000	**DRB1*13**	26	27.66	34	36.17	0.273
**DRB1*07**	15	15.96	15	15.96	1.000	**DRB1*14**	09	9.57	09	9.57	1.000
**DRB1*08**	11	11.70	06	6.38	0.309	**DRB1*15**	28	29.79	23	24.47	0.512
**DRB1*09**	04	4.25	02	2.13	0.682	**DRB1*16**	10	10.64	10	10.64	1.000
**DRB1*10**	02	2.13	07	7.45	0.169						
**DQB1*02**	37	37.23	28	29.79	0.354	**DQB1*05**	38	40.42	45	47.87	0.378
**DQB1*03**	57	60.64	58	61.70	1.000	**DQB1*06**	46	48.94	52	55.32	0.465
**DQB1*04**	12	12.76	05	5.32	0.125						

N: number of individuals, n: number of alleles (2n), %: allele frequency, p: Fisher's exact test ($p \le 0.05$).
