# Supplementary material for: Human leukocyte antigen class I and class II alleles are associated with susceptibility and resistance in borderline leprosy patients from Southeast Brazil
Source: BMC Infect Dis. 2015 Jan 21;15:22. doi: 10.1186/s12879-015-0751-0 (PMC4307149; doi:10.1186/s12879-015-0751-0)
Supplement: Additional file 2: — 2.1 HLA-A*, B*, C*, DRB1* and DQB1* alleles frequencies in 70 BT leprosy and 132 BB and BL leprosy patients controls. 2.2. HLA-A*, B*, C*, DRB1* and DQB1* alleles frequencies in 88 BB leprosy and 114 BT and BL leprosy patients controls. 2.3. HLA-A*, B*, C*, DRB1* and DQB1* alleles frequencies in 44 BL leprosy and 158 BT and BB leprosy patients controls. [file 12879_2015_751_MOESM2_ESM.pdf]

Additional file 2.1. HLA-A*, B*, C*, DRB1* and DQB1* alleles frequencies in 70 BT leprosy and 132 BB and BL leprosy patients controls.

HLA allele	BT leprosy (N=70)		BB+BL (N=132)		p	HLA allele	BT leprosy (N=70)		BB+BL (N=132)		p
	n	Fa (%)	n	Fa (%)			n	Fa (%)	n	Fa (%)	
**A*01**	13	18.57	20	9.85	0.552	**A*31**	04	5.71	09	6.82	1.000
**A*02**	37	52.86	70	53.03	1.000	**A*32**	03	4.28	08	6.06	0.750
**A*03**	19	27.14	31	23.48	0.608	**A*33**	08	11.43	04	3.03	0.025
**A*11**	05	7.14	17	12.88	0.244	**A*34**	00	0.00	02	1.51	0.544
**A*23**	04	5.71	10	7.57	0.774	**A*36**	01	1.43	01	0.76	1.000
**A*24**	13	18.57	27	20.45	0.853	**A*66**	02	2.86	00	0.00	0.119
**A*25**	02	2.86	01	0.76	0.276	**A*68**	08	11.43	17	12.88	0.826
**A*26**	05	7.14	14	10.61	0.613	**A*69**	00	0.00	00	0.00	1.000
**A*29**	04	5.71	12	9.09	0.585	**A*74**	02	2.86	03	2.27	1.000
**A*30**	10	14.28	17	12.88	0.829	**A*80**	00	0.00	01	0.76	1.000
**B*07**	15	21.43	23	17.42	0.570	**B*47**	01	1.43	01	0.76	1.000
**B*08**	10	14.28	15	11.36	0.654	**B*48**	01	1.43	00	0.00	0.346
**B*13**	03	4.28	04	3.03	0.695	**B*49**	00	0.00	02	1.51	0.544
**B*14**	08	11.43	12	9.09	0.625	**B*50**	01	1.43	03	2.27	1.000
**B*15**	16	22.85	24	18.18	0.460	**B*51**	16	22.85	19	14.39	0.170
**B*18**	11	15.71	14	10.61	0.369	**B*52**	02	2.86	05	3.79	1.000
**B*27**	00	0.00	04	3.03	0.300	**B*53**	06	8.57	08	6.06	0.564
**B*35**	14	20.0	30	22.73	0.722	**B*54**	00	0.00	00	0.00	1.000
**B*37**	02	2.86	03	2.27	1.000	**B*55**	02	2.86	01	0.76	0.276
**B*38**	02	2.86	07	5.30	0.721	**B*56**	00	0.00	01	0.76	1.000
**B*39**	06	8.57	09	6.82	0.778	**B*57**	03	4.28	07	5.30	1.000
**B*40**	02	2.86	21	15.91	0.014	**B*58**	02	2.86	10	7.57	0.223
**B*41**	00	0.00	02	1.51	0.544	**B*59**	00	0.00	00	0.00	1.000
**B*42**	03	4.28	03	2.27	0.419	**B*73**	00	0.00	00	0.00	1.000
**B*44**	09	12.86	26	19.70	0.247	**B*78**	00	0.00	00	0.00	1.000
**B*45**	02	2.86	08	6.06	0.499	**B*81**	03	4.28	02	1.51	0.343
**C*01**	02	2.86	06	4.54	0.716	**C*08**	09	12.85	16	12.12	1.000
**C*02**	04	5.71	14	10.61	0.306	**C*12**	08	11.43	23	17.42	0.309
**C*03**	17	24.28	35	26.51	0.865	**C*14**	04	5.71	04	3.03	0.452
**C*04**	24	34.28	38	28.79	0.427	**C*15**	10	14.28	10	7.57	0.142
**C*05**	03	4.28	09	6.82	0.549	**C*16**	09	12.86	12	9.09	0.469
**C*06**	09	12.85	27	20.45	0.246	**C*17**	04	5.71	04	3.03	0.452
**C*07**	35	50.00	64	48.48	0.883	**C*18**	02	2.86	02	1.51	0.610
**DRB1*01**	11	15.71	26	19.70	0.568	**DRB1*11**	15	21.43	40	30.30	0.188
**DRB1*03**	20	28.57	21	15.91	0.035	**DRB1*12**	02	2.86	02	1.51	0.610
**DRB1*04**	11	15.71	26	19.70	0.568	**DRB1*13**	26	37.14	39	29.54	0.273
**DRB1*07**	12	17.14	21	15.90	0.843	**DRB1*14**	05	7.14	14	10.61	0.613
**DRB1*08**	07	10.00	13	9.85	1.000	**DRB1*15**	17	24.28	39	29.54	0.509
**DRB1*09**	04	5.71	02	1.51	0.185	**DRB1*16**	09	12.86	13	9.85	0.635
**DRB1*10**	01	1.43	08	6.06	0.167						
**DQB1*02**	30	42.85	37	28.03	0.048	**DQB1*05**	30	42.85	62	46.97	0.656
**DQB1*03**	36	51.43	82	62.12	0.177	**DQB1*06**	37	52.85	71	53.79	1.000
**DQB1*04**	07	10.00	12	9.09	0.805						

N: number of individuals, n: number of alleles (2n); %: allele frequency, p: Fisher's exact test $p \leq 0.05$.

Additional file 2.2. HLA-A*, B*, C*, DRB1* and DQB1* alleles frequencies in 88 BB leprosy and 114 BT and BL leprosy patients controls.

HLA allele	BB leprosy (N=88)		BT+BL (N=114)		p	HLA allele	BB leprosy (N=88)		BT+BL (N=114)		p
	n	Fa (%)	n	Fa (%)			n	Fa (%)	n	Fa (%)	
**A*01**	17	19.31	16	14.03	0.341	**A*31**	07	7.95	06	5.26	0.565
**A*02**	44	50.00	63	55.26	0.479	**A*32**	04	4.54	07	6.14	0.759
**A*03**	21	23.86	29	25.44	0.870	**A*33**	03	3.41	09	7.89	0.236
**A*11**	14	15.90	08	7.02	0.066	**A*34**	02	2.27	00	0.00	0.188
**A*23**	04	4.54	10	8.77	0.277	**A*36**	00	0.00	02	1.75	0.505
**A*24**	19	21.59	21	18.42	0.597	**A*66**	00	0.00	02	1.75	0.505
**A*25**	01	1.14	02	1.75	1.000	**A*68**	13	14.77	12	10.52	0.394
**A*26**	10	11.36	09	7.89	0.465	**A*69**	00	0.00	00	0.00	1.000
**A*29**	05	5.68	11	9.65	0.431	**A*74**	01	1.14	04	3.51	0.389
**A*30**	11	12.50	16	14.03	0.836	**A*80**	00	0.00	01	0.88	1.000
**B*07**	13	14.77	25	21.93	0.209	**B*47**	01	1.14	01	0.88	1.000
**B*08**	12	13.64	13	11.40	0.670	**B*48**	00	0.00	01	0.88	1.000
**B*13**	02	2.27	05	4.38	0.701	**B*49**	00	0.00	02	1.75	0.505
**B*14**	10	11.36	10	0.88	0.636	**B*50**	01	1.14	03	2.63	0.633
**B*15**	13	14.77	27	23.68	0.154	**B*51**	12	13.64	23	20.17	0.263
**B*18**	10	11.36	15	13.16	0.830	**B*52**	05	5.68	02	1.75	0.243
**B*27**	04	4.54	00	0.00	0.094	**B*53**	04	4.54	10	8.77	0.277
**B*35**	21	23.86	23	20.17	0.606	**B*54**	00	0.00	00	0.00	1.000
**B*37**	03	3.41	02	1.75	0.654	**B*55**	01	1.14	02	1.75	1.000
**B*38**	05	5.68	04	3.51	0.507	**B*56**	01	1.14	00	0.00	1.000
**B*39**	04	4.54	11	9.65	0.189	**B*57**	05	5.68	05	4.38	0.749
**B*40**	13	14.77	10	8.77	0.376	**B*58**	10	11.36	02	1.75	0.012
**B*41**	02	2.27	00	0.00	0.188	**B*59**	00	0.00	00	0.00	1.000
**B*42**	03	3.41	03	2.63	1.000	**B*73**	00	0.00	00	0.00	1.000
**B*44**	16	18.18	19	16.67	0.852	**B*78**	00	0.00	00	0.00	1.000
**B*45**	04	4.54	06	5.26	1.000	**B*81**	01	1.14	04	3.51	0.389
**C*01**	05	5.68	03	2.63	0.299	**C*08**	12	13.64	13	11.40	0.670
**C*02**	07	7.95	11	9.64	0.805	**C*12**	19	21.59	12	10.53	0.033
**C*03**	20	22.73	32	28.07	0.420	**C*14**	00	0.00	08	7.02	0.070
**C*04**	25	28.41	37	32.45	0.644	**C*15**	08	9.09	12	10.53	0.815
**C*05**	05	5.68	07	6.14	1.000	**C*16**	07	7.95	14	12.28	0.360
**C*06**	21	23.86	15	13.16	0.063	**C*17**	04	4.54	04	3.51	0.730
**C*07**	41	46.59	58	50.88	0.572	**C*18**	02	2.27	02	1.75	1.000
**DRB1*01**	14	15.91	23	20.17	0.468	**DRB1*11**	26	29.54	29	25.44	0.527
**DRB1*03**	17	19.32	24	21.05	0.860	**DRB1*12**	01	1.14	03	2.63	0.633
**DRB1*04**	18	20.45	19	16.67	0.590	**DRB1*13**	25	28.41	40	35.09	0.363
**DRB1*07**	16	18.18	17	14.91	0.568	**DRB1*14**	10	11.36	09	7.89	0.469
**DRB1*08**	06	6.82	14	12.28	0.239	**DRB1*15**	26	29.54	30	26.31	0.637
**DRB1*09**	01	1.14	05	4.38	0.235	**DRB1*16**	10	11.36	12	10.53	1.000
**DRB1*10**	06	6.82	03	2.63	0.181						
**DQB1*02**	29	32.95	38	33.33	1.000	**DQB1*05**	41	46.59	51	44.74	0.886
**DQB1*03**	54	61.36	64	56.14	0.474	**DQB1*06**	45	51.14	63	55.26	0.572
**DQB1*04**	07	7.95	12	10.53	0.630						

N: number of individuals, n: number of alleles (2n); %: allele frequency, p: Fisher's exact test $p \leq 0.05$.

Additional file 2.3. HLA-A*, B*, C*, DRB1* and DQB1* alleles frequencies in 44 BL leprosy and 158 BT and BB leprosy patients controls.

HLA allele	BL leprosy (N=44)		BT+BB (N=158)		p	HLA allele	BL leprosy (N=44)		BT+BB (N=158)		p
	n	Fa (%)	n	Fa (%)			n	Fa (%)	n	Fa (%)	
**A*01**	03	6.82	30	18.99	0.064	**A*31**	02	4.54	11	6.96	0.737
**A*02**	26	59.09	81	61.36	0.396	**A*32**	04	9.09	07	4.43	0.259
**A*03**	10	22.73	40	30.30	0.844	**A*33**	01	2.27	11	6.96	0.469
**A*11**	03	6.82	19	14.40	0.420	**A*34**	00	0.00	02	1.26	1.000
**A*23**	06	13.64	08	6.06	0.084	**A*36**	01	2.27	01	0.63	0.389
**A*24**	08	18.18	32	20.25	0.833	**A*66**	00	0.00	02	1.26	1.000
**A*25**	00	0.00	03	1.90	1.000	**A*68**	04	9.09	21	13.29	0.607
**A*26**	04	9.09	15	9.50	1.000	**A*69**	00	0.00	00	0.00	1.000
**A*29**	07	15.91	09	5.70	0.051	**A*74**	02	4.54	03	1.90	0.298
**A*30**	06	13.64	21	13.29	1.000	**A*80**	01	2.27	00	0.00	0.217
**B*07**	10	22.73	28	17.72	0.513	**B*47**	00	0.00	02	1.26	1.000
**B*08**	03	6.82	22	13.92	0.300	**B*48**	00	0.00	01	0.63	1.000
**B*13**	02	4.54	05	3.16	0.647	**B*49**	02	4.54	00	0.00	0.046
**B*14**	02	4.54	18	11.39	0.256	**B*50**	02	4.54	02	1.26	0.207
**B*15**	11	25.00	29	18.35	0.392	**B*51**	07	15.91	28	17.72	1.000
**B*18**	04	9.09	21	13.29	0.607	**B*52**	00	0.00	07	4.43	0.350
**B*27**	00	0.00	04	2.53	0.578	**B*53**	04	9.09	10	6.33	0.510
**B*35**	09	20.45	35	22.15	1.000	**B*54**	00	0.00	00	0.00	1.000
**B*37**	00	0.00	05	3.16	0.587	**B*55**	00	0.00	03	1.90	1.000
**B*38**	02	4.54	07	4.43	1.000	**B*56**	00	0.00	01	0.63	1.000
**B*39**	05	11.36	10	6.33	0.326	**B*57**	02	4.54	08	5.06	1.000
**B*40**	08	18.18	15	9.49	0.114	**B*58**	00	0.00	12	7.59	0.072
**B*41**	00	0.00	02	1.26	1.000	**B*59**	00	0.00	00	0.00	1.000
**B*42**	00	0.00	06	3.80	0.343	**B*73**	00	0.00	00	0.00	1.000
**B*44**	10	22.73	25	18.82	0.366	**B*78**	00	0.00	00	0.00	1.000
**B*45**	04	9.09	06	3.80	0.229	**B*81**	01	2.27	04	2.53	1.000
**C*01**	01	2.27	07	4.43	1.000	**C*08**	04	9.09	21	13.29	0.607
**C*02**	07	15.91	11	6.96	0.076	**C*12**	04	9.09	27	17.09	0.241
**C*03**	15	34.09	37	23.42	0.173	**C*14**	04	9.09	04	2.53	0.070
**C*04**	13	29.54	49	31.01	1.000	**C*15**	02	4.54	18	11.39	0.256
**C*05**	04	9.09	08	6.06	0.297	**C*16**	05	11.36	16	10.13	0.783
**C*06**	06	13.64	30	18.99	0.507	**C*17**	00	0.00	08	6.06	0.204
**C*07**	23	52.27	76	48.10	0.733	**C*18**	00	0.00	04	2.53	0.578
**DRB1*01**	12	27.27	25	18.82	0.120	**DRB1*11**	14	31.82	41	25.95	0.448
**DRB1*03**	04	9.09	37	23.41	0.044	**DRB1*12**	01	2.27	03	1.90	1.000
**DRB1*04**	08	18.18	29	18.35	1.000	**DRB1*13**	14	31.82	51	32.28	1.000
**DRB1*07**	05	11.36	28	17.72	0.365	**DRB1*14**	04	9.09	15	9.49	1.000
**DRB1*08**	07	15.91	13	8.23	0.153	**DRB1*15**	13	29.54	43	27.21	0.849
**DRB1*09**	01	2.27	05	3.16	1.000	**DRB1*16**	03	6.82	19	12.02	0.420
**DRB1*10**	02	4.54	07	4.43	1.000						
**DQB1*02**	8	18.18	59	37.34	0.020	**DQB1*05**	21	47.73	71	44.94	0.864
**DQB1*03**	28	63.64	90	56.96	0.490	**DQB1*06**	26	59.09	82	51.90	0.494
**DQB1*04**	5	11.36	14	8.86	0.570						

N: number of individuals, n: number of alleles (2n); %: allele frequency, p: Fisher's exact test $p \leq 0.05$.
